# Experimental–Analytical Method for Determining the Dynamic Coefficients of Turning Tools

**DOI:** 10.3390/ma18030563

**Published:** 2025-01-26

**Authors:** Lukasz Nowakowski, Slawomir Blasiak, Michal Skrzyniarz, Jaroslaw Rolek

**Affiliations:** 1 Department of Machine Design and Machining, Kielce University of Technology, 25-314 Kielce, Poland; lukasn@tu.kielce.pl (L.N.); mskrzyniarz@tu.kielce.pl (M.S.); 2 Department of Industrial Electrical Engineering and Automatic Control, Kielce University of Technology, 25-314 Kielce, Poland; jrolek@tu.kielce.pl

**Keywords:** tools vibration, silent tools, damping and stiffness coefficients, surface roughness

## Abstract

The article presents an analytical and experimental method for determining the dynamic coefficients of cutting tools, with particular emphasis on turning tools. The method involves aligning the acceleration profile obtained from empirical investigations with a mathematical model describing the oscillations of the cutting tool tip. The stiffness (*k*) and damping (*c*) coefficients determined using this approach enable the design of tools with desired dynamic characteristics, tailored to specific machining processes, such as machining with long overhangs. From the perspective of mechanical dynamics, selecting appropriate stiffness and damping values allows for the design of tools with optimal dynamic properties. High stiffness reduces the occurrence of deformations under external forces, while adequate damping facilitates the rapid attenuation of vibrations, thereby minimising their adverse effects on the machining process. The developed method could serve as a practical tool for identifying the dynamic parameters applicable to a wide variety of cutting tools. The analysis includes three types of turning tools: one with a steel shank, another with a carbide-core steel shank, and a third with a carbon fibre-core steel shank. The results of the tests indicate that the E-A20Q SDUCL 11 tool is best suited for operations requiring high stability and minimal vibration, owing to its favourable damping and stiffness properties.

## 1. Introduction

The objective of surface-shaping technologies, including turning, milling, drilling, reaming [1,2], and broaching [3], as well as electro-erosive or ultrasonic machining [4,5], is to achieve a precise dimensional accuracy and the desired geometric structure of the surface [6]. The cutting process is inherently complex, as it is accompanied by a multitude of phenomena of varying natures, i.e., chip formation and its subsequent plastic deformation, the generation of cutting forces, the presence of residual stresses, thermal phenomena [7], the wear of cutting tools or widely understood vibrations. Furthermore, the aforementioned factors determine the parameters of the geometric structure of the surface, which is obtained during the cutting [8,9] of workpieces. Focusing primarily on tool-to-workpiece vibrations leads to the conclusion that vibrations occurring during machining have a significant impact on the efficiency of the process [10]. Consequently, it is of paramount importance to identify effective methods for reducing these vibrations in production processes. This issue is of particular significance when machining deep holes [11], particularly when using arbour for reaming, deep turning or [12] long overhang milling. The utilisation of tools with substantial overhangs is frequently imperative when machining surfaces or cavities within the workpiece at considerable depth, which often results in an increase in deflection of the cutting tools employed, generating vibrations of higher amplitude. This, in turn, has a detrimental impact on the quality of the machined surfaces, while simultaneously reducing the lifespan of the tools and limiting the overall productivity of the process. It is therefore essential to identify the phenomena associated with the dynamics of cutting tools during machining to improve the efficiency and reliability of the processes under analysis [13]. The concept of stiffness can be examined from two distinct perspectives: static stiffness and dynamic stiffness. The term ‘static stiffness’ is used to describe the ability of a turning tool to resist bending forces under static conditions such as force perpendicular to the main axis of the tool. In general, a structure (construction) which exhibits reduced deformation under the influence of an external load is considered to have higher stiffness. The second type of stiffness—dynamic stiffness, pertains to the capacity of a turning tool to mitigate the impact of oscillating forces or vibrations that arise within the tool-to-workpiece system. This feature is of paramount importance for a turning tool, as it serves as a gauge of its capacity to mitigate vibrations that arise during the machining process which is largely contingent upon its length to diameter ratio and cross-section [14]. In internal turning, the occurrence of vibration is a direct result of the dynamic stability of the tool and clamping system, rather than being solely dependent on the geometrical characteristics of the workpiece. In cases where the clamping system has sufficient rigidity, the primary factor determining the deflection of the tool tip is the ratio of the length of the turning tool overhang (L) to its diameter (D). The dynamic stiffness of such a tool decreases as the distance between the handle and the tip of the turning tool (L) increases and its diameter (D) decreases. This causes mechanical vibrations, which in turn have an adverse effect on the geometric structure of the machined surfaces [15,16], reducing the durability of the cutting edge and the lifespan of the tool. As the overhang increases under stable machining conditions, the amplitude of the vibrations also increases. Nevertheless, modifying the turning tool design by introducing a “damping effect”, in the form of an additional damping mass, may increase the machining capacity at higher overhangs. The “damping effect” reduces the amplitude of the oscillation vibrations over time, thereby suggesting the potential for increasing the dynamic stiffness of the structurally modified cutting tool. As stated previously, the deflection value of the turning tool at the tip of the turning tool results from the cutting forces acting upon it. The deflection value of the turning tool is mainly due to the relationship of the turning tool overhang and its cross-section, but other factors must be taken into account, i.e., the material of the boring bar, the method of its attachment in the tool holder and the direction of the cutting forces.

In recent years, a number of scientific papers have been published with the objective of studying and analysing the dynamic stability of internal turning tools, with a particular focus on those with large overhangs. Many studies have included analytical, numerical and experimental methods to model and predict the dynamic behaviour of a tool under various machining conditions and geometry. However, there is still a lack of consensus on the most appropriate method for assessing the dynamic stability of the tool and its relationship to vibration during machining [17]. Urbikain, in his work [18], suggested that stability diagrams combined with finite element analysis are effective in taking into account the dynamic performance of the machining tool for a specific machining operation, and can contribute to reducing vibration during machining. The author presents two models based on multiple degree of freedom (DOF) analysis to obtain stability plots for heavy-duty turning processes that can evaluate and select the most efficient machine tool, tool holder, and cutting tool for a specific operation. These models can be helpful in assessing the stability limits for a number of input parameters, such as modal data, cutting factors, and the cutting-edge angle of the tool. The article suggests that stability analysis can help improve productivity and reduce vibrations during machining. Several design solutions have been proposed in other works, including dynamic vibration dampers (DVA) [19] and new composite materials for boring bodies [20], which can be used to dampen vibrations during machining. Another approach was to determine the stability criterion according to Nyquist [21], which was used to predict the stability and instability of vibrations during steel treatment. In addition [22], a solution in the form of lateral piezoelectric damping was used in the work, and [23] a tuned mass damper (TMD) was implemented in the work. The authors [24] applied the damping method using hybrid copper and zinc particles positioned in the tool to eliminate self-excited oscillations. Dynamic vibration absorbers (DVA) can be used to absorb vibration energy to increase the efficiency of the operations. In this context, the damping oil inside the empty cylinder in DVA constitutes mean damping, and parameters such as cylinder length, inner, and outer diameters, oil pressure, and excitation frequency are examined to determine their effect on the vibration damping value [25,26].

The presented article contains an analytical and experimental method of determining the dynamic coefficients of cutting tools for turning tools. This method consists of aligning the acceleration course determined through bench tests carried out with a mathematical model describing the vibrations of the turning tool tip. The *k* and *c* coefficients determined in this way will allow us to design tools with the desired dynamic properties adapted to the characteristics of the machining, e.g., long overhang machining.

The main objective of the presented work was to develop an analytical and experimental method for determining the dynamic coefficients of cutting tools used in turning operations. The method is based on the matching of the acceleration waveform obtained during bench tests to a mathematical model describing the vibrations of a turning tool blade. In this way it is possible to determine the stiffness (*k*) and damping (*c*) coefficients that characterise the tools under specific operating conditions. The determination of these parameters will allow the design of tools with specific dynamic properties, which can be important in the case of turning operations with a large overhang of the cutting tool. The method developed aims to better adapt tools to specific machining conditions, minimising the risk of generating vibrations and thus reducing the surface roughness of workpieces. The research also aims to increase the efficiency of machining processes by improving the stability of cutting tools.

## 2. Research Object

The tests involved three types of turning tools labelled as A20Q SDUCL 11, C-A20Q SDUCL 11, and E-A20Q SDUCL 11 manufactured by Maier as tools intended for machining. These tools are classified within a group of folding turning tools with replaceable inserts, which allows for precise and efficient processing of various materials, especially in the processes of boring internal surfaces (holes).

**Figure 1:**
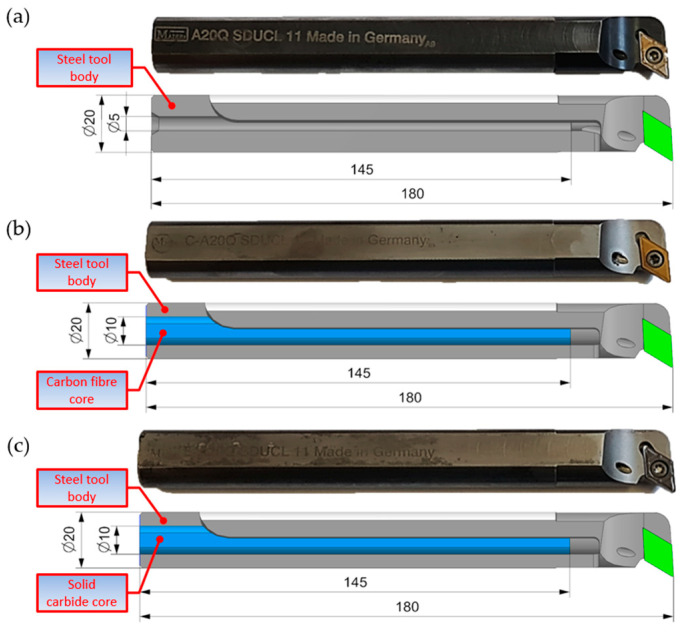
Turning tools: (**a**) A20Q SDUCL 11 turning tool, (**b**) C-A20Q SDUCL 11 turning tool, (**c**) E-A20Q SDUCL 11 turning tool.

Figure 1a shows turning tool A20Q SDUCL 11, which has a monolithic construction with a central hole for the supply of coolant and lubrication. The tool is used for standard internal turning operations (boring). Its design is suitable for most materials, such as steel, stainless steel, or cast iron. The C-A20Q SDUCL 11 (Figure 1b) turning tool has a hybrid design in which a carbon fibre core with a diameter of 10 mm and a length of 145 mm is embedded in the steel tool body. The tool is fitted with a duct for feeding the coolant directly to the cutting insert, making it suitable for demanding applications such as stainless steel or titanium alloys, where high operating temperatures may affect tool durability. The cooling system allows the extension of the lifespan of the cutting insert and enhances the quality of the machined surface. Whereas the E-A20Q SDUCL 11 (Figure 1c), thanks to the steel shank in which a solid carbide core with a diameter of 10 mm and 145 mm is embedded, is dedicated to the most demanding turning operations, where high stiffness and resistance to vibrations occurring in the tool–workpiece system is necessary.

The chemical composition of the materials (Table 1) from which the tested turning tools were made was analysed using X-ray fluorescence (XRF) on an Olympus Vanta VCR handheld analyser (OLIMPUS-IMS, Evident, USA).

Due to its mechanical properties, sintered carbide provides greater tool strength in difficult cutting conditions, especially when machining materials with high hardness. Table 2 shows the weights of specific components for the cutting tools tested, which were determined using the RADWAG laboratory scale WLC 1/A2/C/2.

Analysis of the assembly of the tool body, the cutting plate, the plate mounting bolt, and the tool holder leads to the conclusion that the kit containing the tool labelled as E-A20Q SDUCL 11 has the heaviest mass of 1.828 kg thanks to the shank made of steel with a carbide core. The smallest tool with the symbol C-A20Q SDUCL 11 had a total weight of 1.683 kg with a carbon fibre core. An increase in the mass of the set can result in an enhancement of the dynamic system’s inertia, which may ultimately lead to a reduction in the resonance frequency. A higher mass of the oscillatory system may be more effective in suppressing vibration, which requires precise fine tuning of the vibration damping parameters.

## 3. Results

The mathematical model of the turning tool is presented in a manner similar to the case of a unilaterally fixed beam, which can be described as a second-order ordinary differential equation. Such an equation takes into account the weight of the tool and the stiffness and damping factors. The model also assumes that the tip of the cutting insert of the turning tool generates vibrations in a single direction (deflection in the vertical plane). The equation describing the vibrations caused by the turning tool may be presented in the following, widely known manner:

(1) $$m\;\ddot{x}\left(t\right)\;+c\;\dot{x}\left(t\right)+k\;x\left(t\right)=F\left(t\right)$$

where

*m*—effective mass of the tool (kg);
*c*—damping coefficient, which models motion resistance (e.g., friction, material damping) (N/ms);
*k*—stiffness factor that models tool elasticity, (N/m);
*x(t)*—the displacement (deflection) of the tip of the turning tool as a function of time (m);
*F(t)*—external force (cutting) acting on the tool, as a function of time (N).

The solution of this equation was made using the Laplace transform and the Green character function

(2) $$y\left(t\right)=\int_{0}^{t}G\left(t-\tau \right)\;f\left(\tau \right)d\tau$$

and initial conditions $\dot{x}\left(0\right)=0,\;\;x\left(t\right)=0$.

The final form of the dependency describing the displacement of the tip of the insert is as follows:

(3) $$x\left(t\right)=\frac{1}{m}\frac{1}{\sqrt{\lambda^{2}-b^{2}}}\int_{0}^{t}e^{-b\left(t-\tau \right)}\sin\left(\sqrt{\lambda^{2}-b^{2}}\left(t-\tau \right)\right)F\left(\tau \right)\;d\tau$$

or when the driving force (cutting force) disappears ($F\left(t\right)=0$)

(4) $$x\left(t\right)=e^{-bt}\left(c_{1}\sin\left(\sqrt{\lambda^{2}-b^{2}}t\right)+c_{2}\cos\left(\sqrt{\lambda^{2}-b^{2}}t\right)\right)$$

or after applying specific algebraic transformations, where $A_{0}=\sqrt{c_{1}^{2}+c_{2}^{2}}$, and $\cos\varphi =c_{2}/A_{0}$ result in

(5) $$x\left(t\right)=A_{0}\;e^{-bt}\cos\left(\sqrt{\lambda^{2}-b^{2}}t-\varphi \right)$$

where c/m=2b, $k/m=\lambda^{2}$.

And the second derivative of Equation (5) describes the acceleration of the tip of the insert of the turning tool in the following form:

(6) $$x^{\prime \prime }\left(t\right)=A_{0}\;e^{-bt}\left(2b\sqrt{\lambda^{2}-b^{2}}\sin\left(\sqrt{\lambda^{2}-b^{2}}t-\varphi \right)+\left(2b^{2}-\lambda^{2}\right)\cos\left(\sqrt{\lambda^{2}-b^{2}}t-\varphi \right)\right)$$

**Figure 2:**
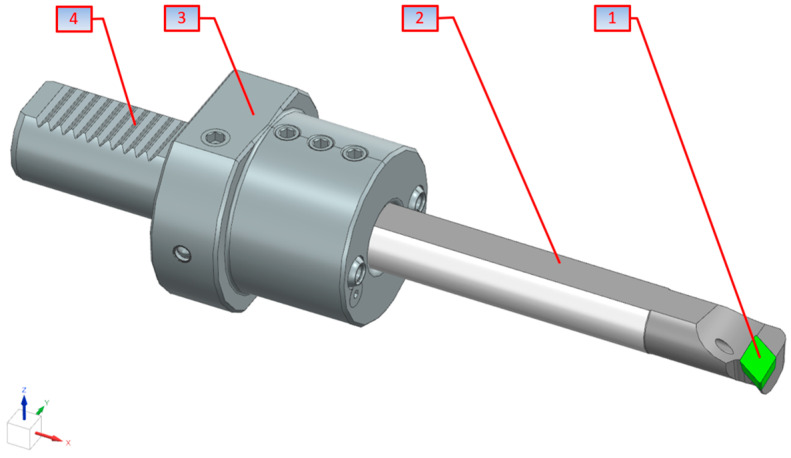
Turning tool: (1) insert, (2) body, (3) tool holder, (4) gripping part of the tool holder.

In such a mathematical approach, a turning tool is treated as a beam of a specific length, where its one end (the tool grip) is rigidly fixed in the holder, while the other end (the working part of the tool) is subjected to a cutting force, which generates vibrations of a specific nature (Figure 2).

It can be assumed that the tool in question consists of two main mass elements, i.e., the cutting tool holder and the tool itself embedded therein.

## 4. Test Rig

**Figure 3:**
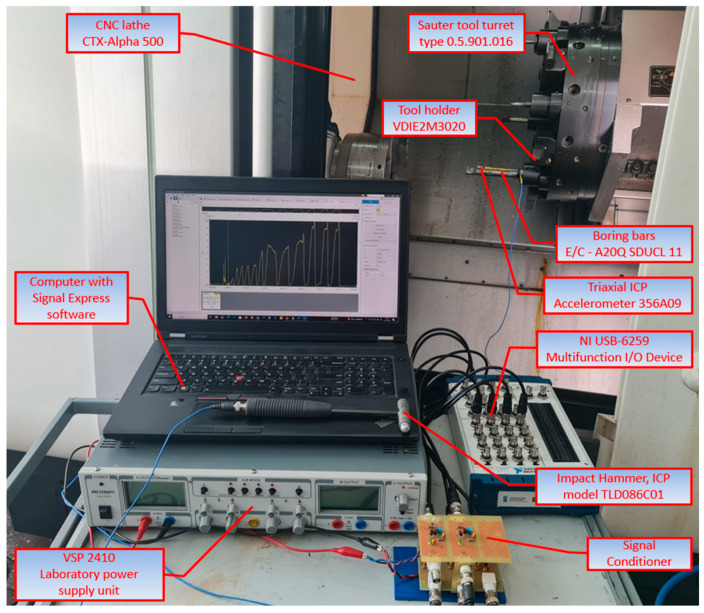
Test stand.

The laboratory tests were carried out on the measuring stand (Figure 3), constructed with the CTX alpha 500 lathe (DMG Mori Co., Ltd., Tortona, Italy) equipped with Sinumeric 840SL control, fitted with a 12-position radial turret manufactured by Sauter, type 0.5.901.016 (Sauter Feinmechanik GmbH, Metzingen, Germany) for the VDI 30 tool holder. Three distinct designs of boring tools manufactured by Maier GmbH have been installed in the turret with the tool holder VDIE2M3020 (Kennametal Inc., Pittsburgh, PA, USA). The tools used included the following:

- A20Q SDUCL 11: A boring bar with a ground shaft and internal coolant.
- C–A20Q SDUCL 11: A boring bar with a carbon fibre core, ground shaft, and internal coolant.
- E–A20Q SDUCL 11: A boring bar with a solid carbide core, ground shaft, and internal coolant.

**Figure 4:**
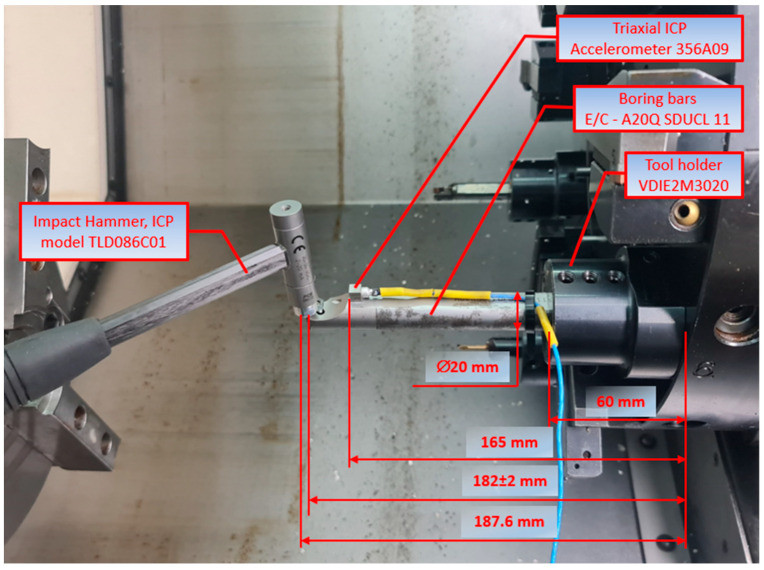
Test stand—the inside of the working space of the machine tool.

Experimental research consisted of the artificial introduction to the system of forcing in the form of an external force with the following model of a modal hammer: TLD086C01 (PCB Piezotronics) (Figure 4). Measurement of the system response on boring bars was taken with the acceleration sensor model 356A09 (Triaxial ICP Accelerometer PCB Piezotronics). The signals from the modal hammer and accelerometer were recorded using the NI USB-6259 (National Instruments) measuring card at a frequency of 10 kHz and Signal Express software (version 2015). A conditioning system consisting of low-pass filters with a cut-off frequency of 5 kHz was used to prevent aliasing in the signal sampling process. Furthermore, the conditioning system incorporated current sources that facilitated the provision of an appropriate supply to the modal hammer and accelerometer.

**Figure 5:**
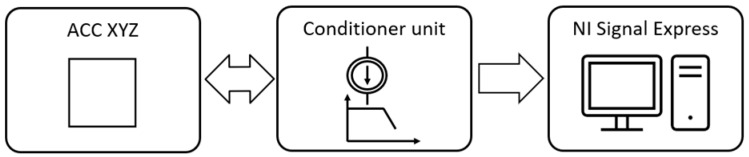
The process of recording the measurement data.

During the course of the experiment, measurement data were recorded according to the procedure shown in Figure 5. The recorded data were then analysed using Matlab software (R2024a).

In order to determine the free constituent of the signal, it was subject to an FFT analysis. The characteristic frequencies of the waveforms have been determined through this method. Removing these frequencies allowed us to obtain the forced component of the signal.

**Figure 6:**
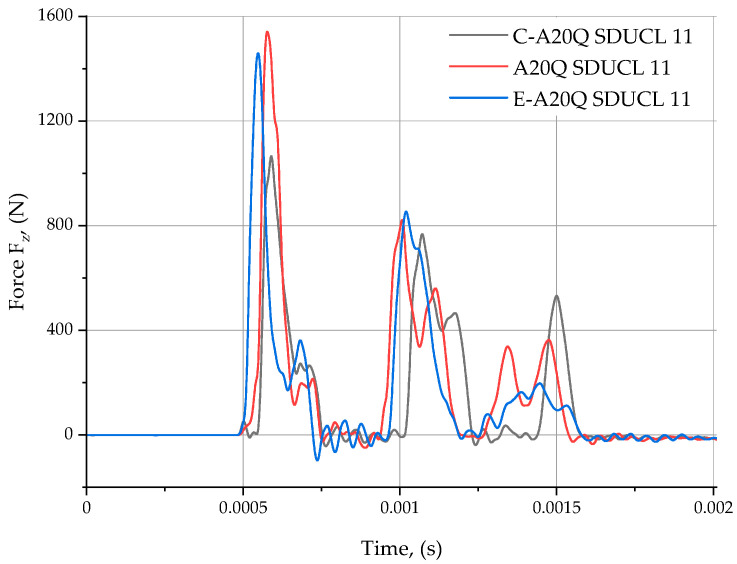
The course of driving forces.

**Figure 7:**
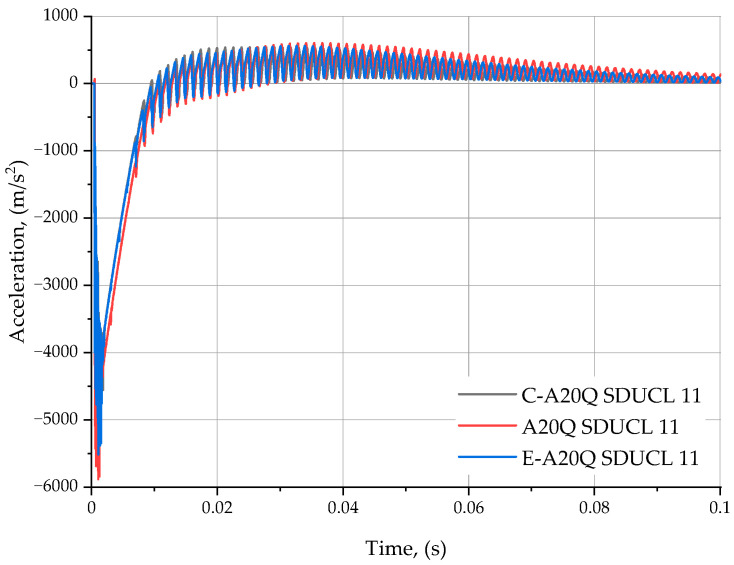
Z-axis acceleration waveforms for pulse forcing.

The obtained results are presented in the graphs below (Figure 6 and Figure 7).

Figure 6 depicts the course of force changes as a function of time, caused by the impact of a modal hammer, for the three types of turning tools examined.

The analysis of the data presented in the graph (Figure 6) allows us to observe that in the case of the monolithic tool A20Q SDUCL 11 (red line), the force reaches the highest peak value of approximately 0.0005 s and its value is close to 1500 N. In the case of the other two measuring series, 11 (the tool with carbon fibre core C-A20Q SDUCL 11 (black line) and the tool with sintered carbide E–A20Q SDUCL 11 (blue line) also display significant peaks at a similar time; however the values are slightly lower. The lowest force value was identified in the case of a carbon-core turning tool at approximately 1050 N. The differences result from the lack of repeatability due to the manual use of a modal hammer by the operator during the tests.

All three measurement series demonstrate a tendency to stabilise at a value close to zero following an interval of approximately 0.0018 s. This suggests the presence of a significant and transient driving force acting upon the test objects. At the outset of the measurement period, the registered forces undergo a phase of pronounced transformation, subsequently attaining a state of equilibrium and approaching a value of negligible magnitude.

The acceleration measurement results are presented graphically on Figure 7.

The graph of acceleration changes as a function of time (Figure 7) in the initial phase and all three waveforms reach their maximum negative acceleration values of −5500 m/s^2^, which indicates the action of a pulsed force opposite the *Z* axis of the adopted coordinate system. Moreover, an evident oscillating effect is discernible on the graph, particularly during the initial phase of the run (up to approximately 0.03 s). These oscillations are characterised by decreasing amplitude, which clearly indicates the dynamic damping process occurring in this system, and thus the dissipation of energy. After the initial and oscillating phases, the system stabilises near a value close to zero, which in turn indicates that the system reaches a state of dynamic equilibrium. The observed variations in acceleration rates for all three types of turning tools tested may be indicative of slight differences in the values of the parameters characterising each of them (e.g., weight, damping coefficient, stiffness).

## 5. Results and Discussion

The Levenberg–Marquardt method was used for the purpose of matching the plots of the theoretical acceleration course of the vibrating tip of the tool to the results of the bench tests. This is one optimisation method, especially used to solve non-linear problems of the smallest squares, in particular in the case of matching equations with data presented in graphs [27]. The method features a synthetic merger of components of gradient and Newtonian methods, which facilitates accelerated convergence and enhanced stability. This method is primarily employed in the process of aligning non-linear models with empirical data, a process that may be applied to the calibration of parameters within a range of physical, chemical, and biological models. Literature indicates that the method is effective in complex models, particularly in cases that require precise matching of parameters such as calibration of spectroscopy in analytical models (e.g., WMS model). In such instances, it guarantees high sensitivity and resistance to changing environmental conditions [28].

Adaptive approaches to the Levenberg–Marquardt method have been developed to improve convergence, primarily in relation to problems posed by large data sets. Yan i et al. (2021) described the Levenberg–Marquardt adaptive algorithm for neural networks, which successfully increases optimisation efficiency by adjusting the damping parameter based on changing target function conditions [29]. However, studies on the convergence of the method indicate improved stability, in particular in industrial application models, such as supply chain optimisation analyses, where the method allows us to obtain the global convergence and decrease the time required for calculations [30].

Examples of the Levenberg–Marquardt method presented above illustrate the extensive potential for its utilisation in a multitude of applications. It was noted that due to its versatility and wide application possibilities, it can be used for adjusting dynamic coefficients of vibration systems, such as the examined turning tools.

The experimental and analytical part of the analyses involved the use of the Levenberg–Marquardt method to match the results obtained from experimental studies, i.e., acceleration courses for the examined lathes and determination of *c* and *k* coefficients in the equation on this basis (1).

**Figure 8:**
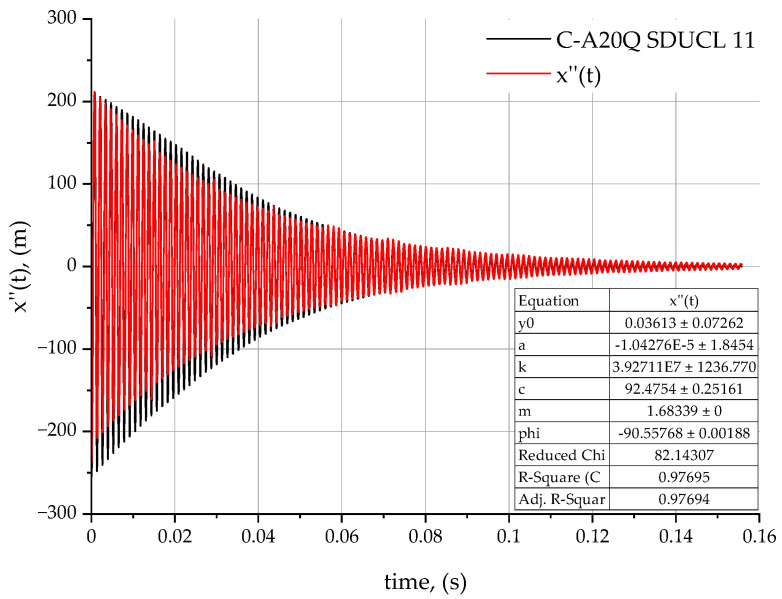
Acceleration rate for C-A20Q SDUCL 11 turning tool.

**Figure 9:**
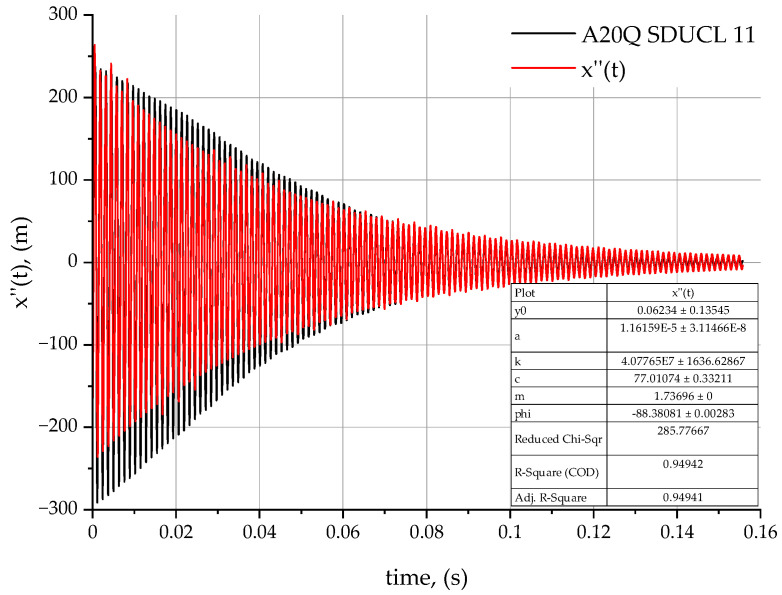
Acceleration rate for the A20Q SDUCL 11 turning tool.

**Figure 10:**
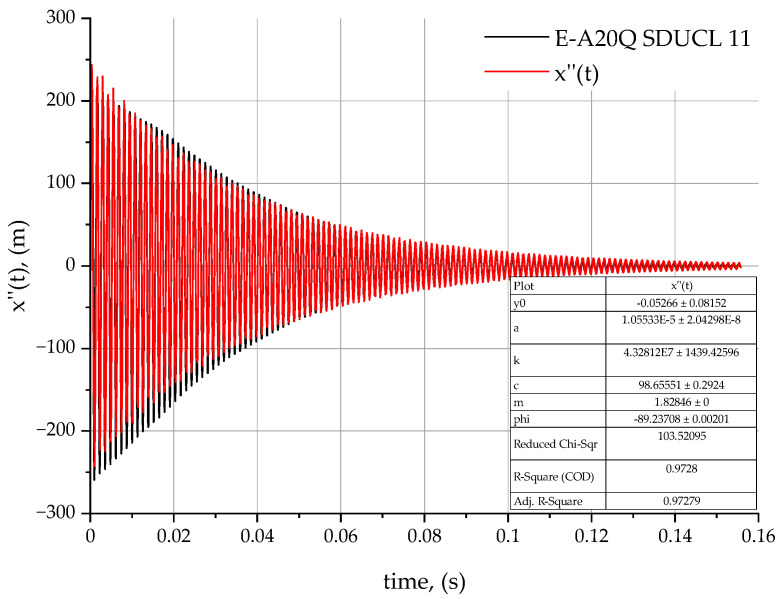
Acceleration rate for the E-A20Q SDUCL 11 turning tool.

The analysis of the results obtained are based on the proposed method of determining the values of dynamic coefficients (damping *c* and stiffness *k*) for the three turning tools tested—C-A20Q SDUCL 11 (Figure 8), A20Q SDUCL 11 (Figure 9), and E-A20Q SDUCL 11 (Figure 10). These factors are fundamental to an understanding of the dynamics of mechanical systems. The damped vibration theory may prove useful for understanding the influence of the design of the turning tool on the dynamic behaviour, stability, and durability of the tool during the machining process.

Stiffness coefficient *k* is a key parameter with an impact on the stability of the entire tool to workpiece system during the machining process. This is related to the resistance of the tool itself to deformation under the influence of external forces, in this case cutting forces. In the case of the C-A20Q SDUCL 11 tool, the stiffness coefficient is $3.927\times 10^{7}$ N/m, which is the lowest value among the analysed tools. The A20Q SDUCL 11 tool features a slightly higher stiffness at $4.077\times 10^{7}$ N/m and the highest stiffness of $4.328\times 10^{7}$ N/m was observed for the E-A20Q SDUCL 11 tool. Higher stiffness coefficients indicate better tool stability and reduced deformation during operation, which can result in enhanced surface quality, particularly at elevated tool overhangs and under dynamic loads.

In the case of the damping coefficient *c*, which has a potential effect on the decay rate (extinction) of vibrations, a difference between the tools under analysis is observable. In the case of the C-A20Q SDUCL 11 turning tool, the *c*-value is 92.475  Ns/m, indicating moderate vibration damping. In the case of the A20Q SDUCL 11 tool, the value is lower at 77.010 Ns/m, while the E-A20Q SDUCL 11 tool has the highest attenuation coefficient—equal to 98.655 Ns/m. Higher values of the damping coefficient *c* contribute to faster vibration decay, which is beneficial for the stability of the system and allows for a more stable treatment process.

Table 3 shows the stiffness and damping factors for the analysed turning tools.

In addition, the stiffness of the system is related to the natural vibration frequency λ expressed as an equation parameter (5), where value λ indicates the frequency at which the system tends to vibrate. The analysis of the natural vibration frequency λ from stiffness leads to a conclusion that tools with a higher value of *k* stiffness factor will have a higher natural frequency, which reduces their susceptibility to resonance at lower forcing frequencies.

**Figure 11:**
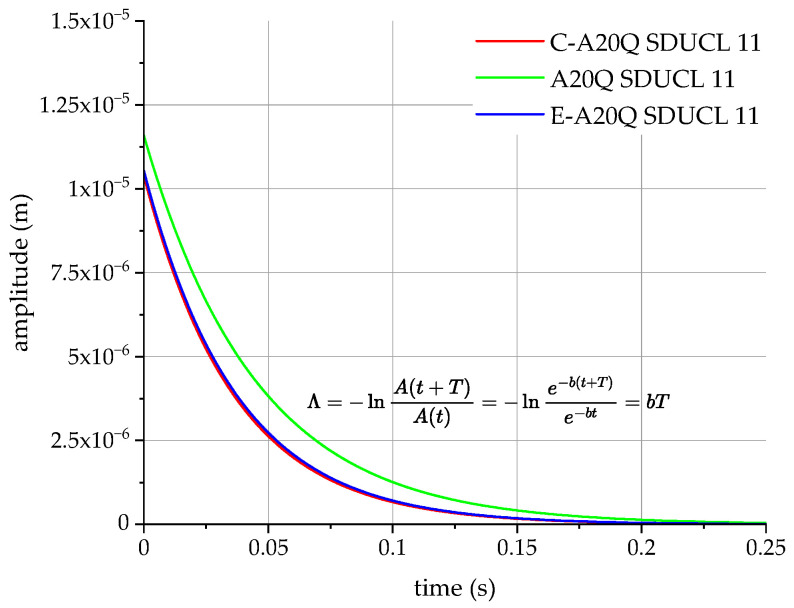
Changes in vibration amplitude over time for the cutting tools tested.

The highest stiffness value in the case of the E-A20Q SDUCL 11 tool means that it is more resistant to deformation and has better dynamic stability but less damping capacity, as can be seen in Figure 11. This means that it will be more useful for working under heavy load conditions as well as in conditions where increased vibrations may occur due to increased cutting forces.

**Figure 12:**
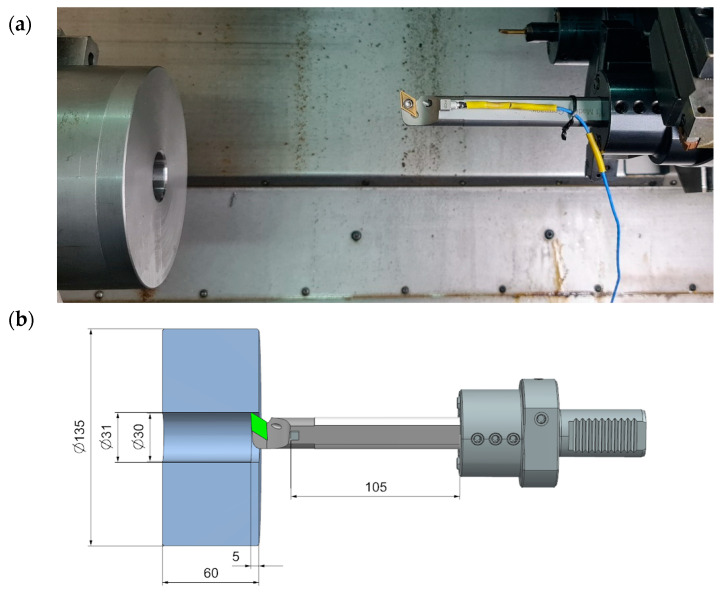
Cutting tests, (**a**) view of the boring process, (**b**) boring diagram.

As part of the research, cutting tests were carried out without the use of coolant lubricant on a CTX ALPHA 500 turning centre from DMG Mori (Tortona, Italy). The field tests involved machining a hole with a diameter of 31 mm and a depth of 5 mm. The semi-finished part was made of S355J2 (1.0570) steel in the form of a sleeve with the following dimensions: outer diameter 135 mm, pre-hole diameter 30 mm, sample height 60 mm. A schematic of the machining process is shown in Figure 12.

**Figure 13:**
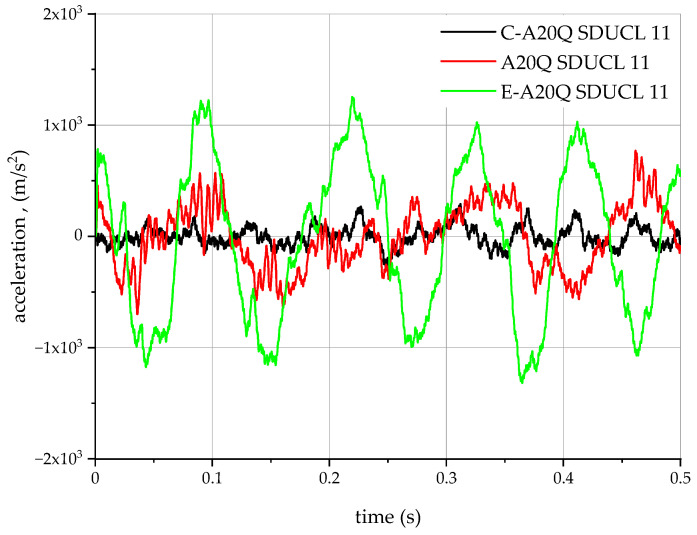
Changes in acceleration during internal lathing.

The hole surface was machined with the following parameters: cutting speed v_c_ = 107 m/min (s = 1100 rpm), feed rate f_n_ = 0.2 mm/rev, depth of cut a_p_ = 0.5 mm. The lathe tools were fitted with a DCMT 11 T3 04-UF 1125 insert with a PVD TiAlN + TiAlN coating from Sandvik Coromant (Sandviken, Sweden). During the cutting process, acceleration waveforms were recorded as a function of time. Accelerations were recorded using the same measuring system as for the modal hammer tests. On this basis, the results were collected and presented graphically in Figure 13.

**Figure 14:**
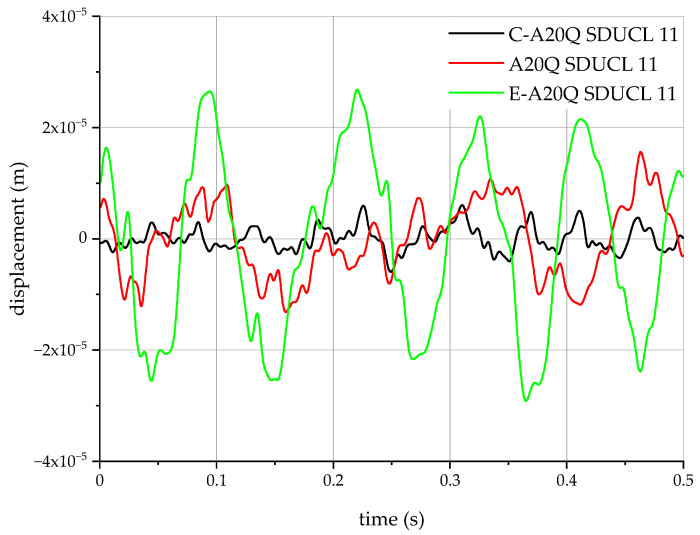
Changes in displacement during internal lathing.

Using the data collected in Table 3 for the cutting tools investigated and the measured accelerations, the displacement of the cutting tool tip were determined. The results obtained are shown in Figure 14.

When analysing the range of vibration amplitudes, it can be observed that the tool designated C-A20Q SDUCL 11 has the smallest vibration amplitude, which is in the range of approximately ±1 × 10^−5^ m. For the A20Q SDUCL 11 tool, the vibration amplitude is significantly larger, oscillating in the range of approximately ±2 × 10^−5^ m. The largest amplitude was observed for the E-A20Q SDUCL 11 tool, reaching values close to ±3 × 10^−5^ m. From this analysis, it can be concluded that the C-A20Q SDUCL 11 tool has the best vibration dampening properties, which is essential for achieving high-quality machined surfaces, minimising wear on the cutting edges and increasing the overall efficiency and life of the cutting tool, thus ensuring more stable and accurate machining processes.

## 6. Conclusions

The experimental and analytical methodology employed in the determination of the dynamic coefficients of turning tools allows for a highly accurate estimation of the fundamental parameters characterising vibration systems. This allows us to state that the combination of a high stiffness and damping coefficient, as exemplified by the E-A20Q SDUCL 11 tool, offers enhanced resilience to internal vibrations and those generated by the tool–workpiece system. Higher *k* and *c* values mean that the turning tool may be less susceptible to resonance, which is of key value in applications requiring high stability. Tools with lower *k* and *c* values, such as A20Q SDUCL 11 tool, may be more susceptible to generated vibrations, which may result in higher surface roughness and increased tool wear.

From the perspective of mechanical system dynamics, the selection of appropriate stiffness and damping values enables the design of tools with the desired dynamic properties. High stiffness serves to diminish the occurrence of deformation under the influence of external forces, whereas adequate damping facilitates the rapid attenuation of vibrations, thereby limiting their detrimental impact on the machining process. In conclusion, the analysis of *c* and *k* coefficients shows that the E-A20Q SDUCL 11 tool displaying the highest values of the parameters in question, is best suited for working in conditions requiring high stability and minimal vibration.

## Figures and Tables

**Table 1:** Chemical composition of cutting tool materials.

Steel Tool Body	Carbon Fibre Core	Solid Carbide Core
Cr: 1.91%	Carbon fibre-reinforced polymers Carbon fibres Epoxy	W: 86.91%
Mn: 1.54%		Co: 10.09%
Mo: 0.15%		Cr: 0.62%
Ni: 0.12%		Mo: 0.25%
Si: 0.40%		Ni: 0.132%

**Table 2:** Weight of the components of the turning tool.

Name	Monolithic Tool	Tool with Carbon Fibre Core	Tool with Carbide Core
Symbol	A20Q SDUCL 11	C-A20Q SDUCL 11	E-A20Q SDUCL 11
Weight, g.	366.37	312.8	457.87
Tool holder mass VDIE2M3020, g	1365	1365	1365
Weight of cutting plate, g	4.86	4.86	4.86
Mass of plate fixing bolt, g	0.73	0.73	0.73
Weight of the set, g	1736	1683	1828

**Table 3:** Dynamic parameters of turning tools.

Tool Type	m [kg]	c [Ns/m]	k [N/m]	Error *c*	Error *k*
C-A20Q SDUCL 11	1.683	92.475	3.927 × 10^7^	0.251	1236.77
A20Q SDUCL 11	1.736	77.010	4.077 × 10^7^	0.332	1636.62
E-A20Q SDUCL 11	1.828	98.655	4.328 × 10^7^	0.292	1439.25

## Data Availability

Data are contained within the article.
